# Growth Factor Quantification of Platelet-Rich Plasma in Burn Patients Compared to Matched Healthy Volunteers

**DOI:** 10.3390/ijms20020288

**Published:** 2019-01-12

**Authors:** Roos E. Marck, Kim L. M. Gardien, Marcel Vlig, Roelf S. Breederveld, Esther Middelkoop

**Affiliations:** 1 Department of Plastic, Reconstructive & Hand Surgery, Amsterdam UMC, University of Amsterdam, 1081 HV Amsterdam, The Netherlands; roosmarck@me.com; 2 Burn Center, Red Cross Hospital, 1942 LE Beverwijk, The Netherlands; kgardien@rkz.nl (K.L.M.G.); breed@kpnplanet.nl (R.S.B.); 3 Department of Plastic, Reconstructive & Hand Surgery, Amsterdam Movement Sciences Research Institute, Amsterdam UMC, Vrije Universiteit Amsterdam, 1081 HV Amsterdam, The Netherlands; 4 Association of Dutch Burn Centers, 1942 LE Beverwijk, The Netherlands; mvlig@burns.nl; 5 Department of Surgery, Leiden University Medical Center, 2333 ZA Leiden, The Netherlands

**Keywords:** platelets, burns, growth factors, platelet rich plasma, quantification

## Abstract

Platelet rich plasma (PRP) is blood plasma with a platelet concentration above baseline. When activated, PRP releases growth factors involved in all stages of wound healing, potentially boosting the healing process. To expand our knowledge of the effectiveness of PRP, it is crucial to know the content and composition of PRP products. In this study, growth factor quantification measurements of PRP from burn patients and gender- and age-matched controls were performed. The PRP of burn patients showed levels of growth factors comparable to those of the PRP of healthy volunteers. Considerable intra-individual variation in growth factor content was found. However, a correlation was found between the platelet count of the PRP and most of the growth factors measured.

## 1. Introduction

Platelet rich plasma (PRP) is produced from blood plasma, and results in a platelet concentration above baseline. When PRP is activated, platelets release their growth factors, such as platelet-derived growth factor (PDGF), fibroblast growth factor (FGF), transforming growth factor β (TGF-β), epidermal growth factor (EGF), and vascular endothelial growth factor (VEGF). These growth factors are involved in all stages of wound healing. Improved wound healing qualities have been attributed to PRP, by the multitude of growth factors delivered to a wound [1].

There are numerous types of PRP products and preparation methods. An even larger number of different application areas exist, varying from sports medicine and orthopedics to chronic and acute wounds, as well as aesthetic applications. These are described in an extensive body of literature; however, most studies advocating the use of PRP comprise case reports and patient cohorts. Randomized controlled trials are rare, and systematic reviews repeatedly fail to show strong conclusive evidence of the effects of PRP [1,2,3]. This could be partly due to the great variability of PRP products, namely, variability in preparation protocols; different composition (with/without leukocytes and fibrin content); variability in platelet baseline count and PRP yield, growth factor content per platelet; and finally activation and application methods. Furthermore, for all the different applications, the most desirable number of platelets and amount of growth factors that should be used are unknown.

Recently, a new classification system for PRP was proposed by Harrison et al., in an effort to systematize studies done on PRP [4]. This paper also emphasizes the importance of quality control of the platelet preparations. To expand our knowledge of the effectiveness of PRP, it is essential to know the content and composition of the PRP products. Growth factor quantification still seems the best type of quality control of PRP, since platelet count and growth factor quantification do not appear to correlate in a consistent way [5,6]. A recent systematic review of commercial PRP separation systems showed a large heterogeneity in the concentrations of platelets, leukocytes, and growth factors [7]. One of the inclusion criteria of this systematic review was that the studies had to investigate ‘healthy volunteers.’ This is somewhat curious, because autologous PRP is mostly applied in patients and not healthy volunteers. An extensive search of the literature was carried out; however, we could not find studies describing the quantification of PRP content in actual patients.

In burn patients, the use of PRP has been ascribed potential positive effects on burn wound healing [1,8]. The current study was part of a recent randomized trial, which failed to show significant added value of PRP in acute burns [9]. In addition to the variables in PRP in general described above, a few more can be added for burn injury. Burn injury has a severe impact on the internal physiology of patients [10], and platelet counts show a distinct pattern post-burn injury, with a nadir at day 3, a peak around day 14, followed by a gradual return to normal values [11], thus affecting the baseline platelet count from which the PRP is produced. Since platelets are the core ingredient of PRP, it is crucial to know if and how the quality of the PRP could be affected by the burn injury. In a recent study, it was found that the platelets in burn patients were not overly activated and remained functional and not deprived of growth factors; however, this was tested in platelets in whole blood samples [12]. The current study investigated the quality of PRP, classified as L-PRPIIB-1 according to the new classification system, which implicates leukocyte-rich PRP, activated before application (II), with a mean platelet count between 900–1700 (B), and prepared with a gravitational centrifugation technique (1) [4], from burn patients compared with age and gender matched healthy controls.

## 2. Results

### 2.1. Demographics and Hematology

**Figure 1:**
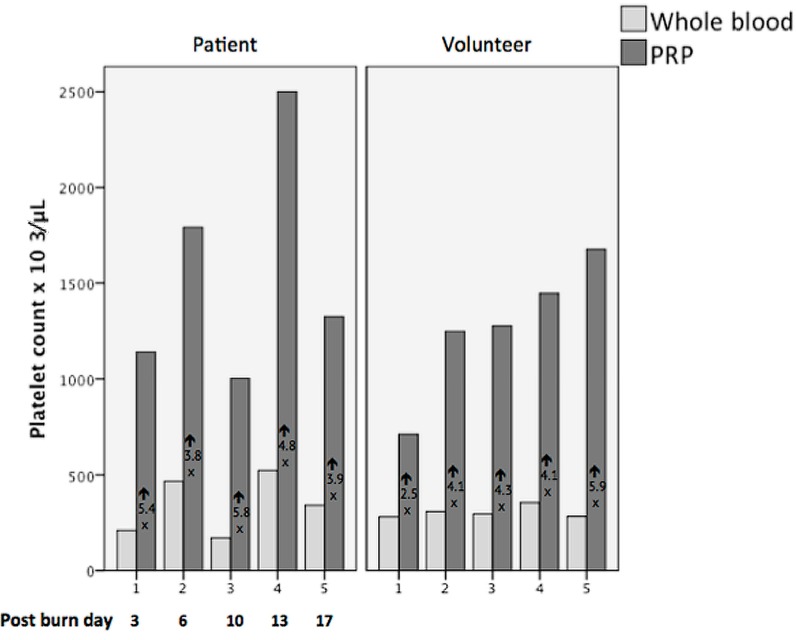
Platelet count in whole blood versus platelet count platelet rich plasma (PRP), in patient group and in volunteer group; the platelet concentration factor is depicted underneath the arrow; under patient group the post- burn day is shown.

The demographics and hematology results are listed in Table 1. By chance, the patients included were sampled on longitudinal days post-burn injury (Figure 1). The platelet counts follow the known time course post-burn injury as has been described, with only patient 3 still having a lower count than expected. A possible explanation could be that this patient suffered the largest burn [11].

### 2.2. Platelet Concentration Ratio

The mean ratio of platelet concentration from whole blood platelet to PRP was 4.44 (SD1.04 range: 2.5 to 5.9) (Figure 1). There was no difference between patients and volunteers, 4.7 vs. 4.2 respectively (*p* = 0.78 Mann–Whitney Test).

### 2.3. Growth Factor Content

No significant difference was found in mean growth factor content between PRP from burn patients and that from matched healthy controls (Mann–Whitney tests: transforming growth factor β (TGFβ-1) (mean patient 57,542 pg/mL vs. volunteers: 45,389 pg/mL) *p* = 0.2; TGFβ-2 (mean patients 1292 pg/mL vs. volunteers 934 pg/mL) *p* = 0.2; TGFβ-3 (mean patients 21 pg/ml vs. volunteers 21 pg/mL) *p* = 0.9; platelet derived growth factor (PDGF-AA) (mean patients 36,327 pg/mL vs. volunteers 32,113 pg/mL) *p* = 0.4; PDGF-BB (mean patients 56,031 pg/mL vs. volunteers 44,566 pg/mL) *p* = 0.5; vascular endothelial growth factor (VEGF) (mean patients 701 pg/mL vs. volunteers 756 pg/mL) *p* = 0.8; epidermal growth factor (EGF) (mean patients 73 pg/mL vs. volunteers 60 pg/mL) *p* = 0.7; fibroblast growth factor (FGF-2) (mean patients 224 pg/mL vs. 220 volunteers pg/mL) *p* = 0.8), nor in growth factor per platelet ratio (data not shown).

**Figure 2:**
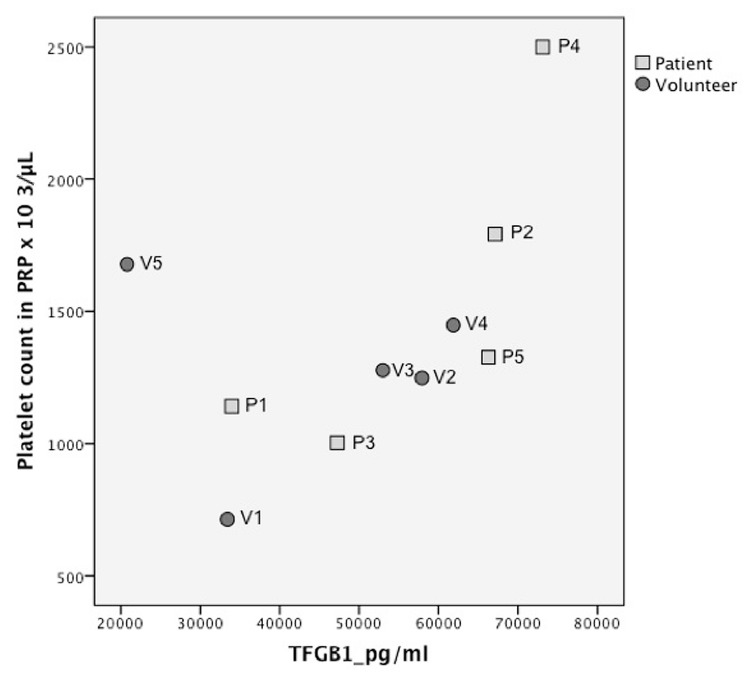
Growth factor quantification per platelet count in platelet rich plasma (PRP) for transforming growth factor β (TGFβ1).

**Figure 3:**
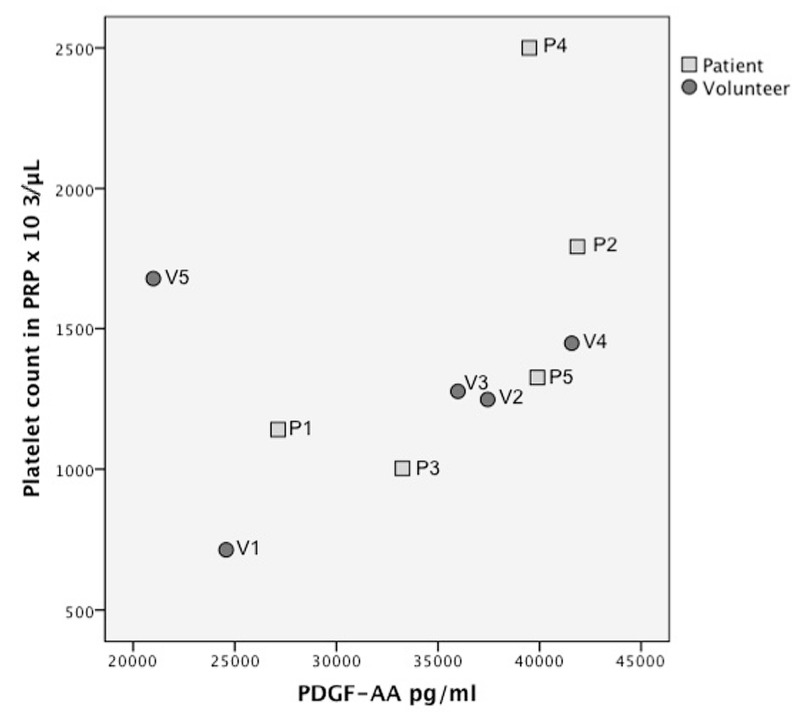
Growth factor quantification per platelet count in platelet rich plasma (PRP) for platelet derived growth factor (PDGF) AA.

**Figure 4:**
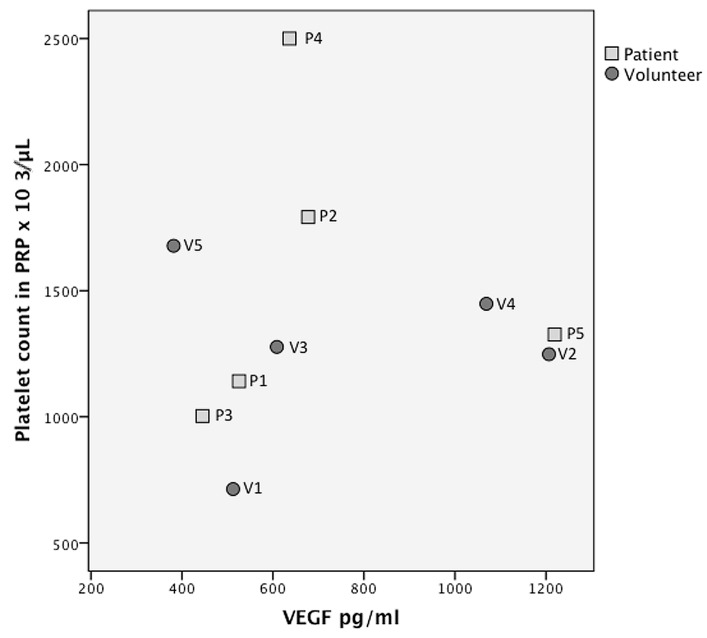
Growth factor quantification per platelet count in platelet rich plasma (PRP) for vascular endothelial growth factor (VEGF).

There was a noticeable correlation between platelets in PRP and growth factor concentration, when volunteer 5 (which was considered an outlier, because it was more than 2SD outside the mean ratio) was eliminated, except for VEGF and FGF (Figure 2, Figure 3 and Figure 4 and Supplemental Figures S1–S5) Spearman’s rho correlation tests: TGFβ1 *R* = 0.95, *p* = 0.008; TGFβ-2 *R* = 0.9, *p* = 0.001; TGFβ-3 *R* = 0.8 *p* = 0.02; PDGF-AA *R* = 0.9, *p* = 0.002; PDGF-BB *R* = 0.8, *p* = 0.008; VEGF *R* = 0.3, *p* = 0.4; FGF-2 *R* = 0.1, *p* = 0.7; EGF *R* = 0.7, *p* = 0.03).

## 3. Discussion

In this study it was shown that PRP of burn patients, L-PRPIIB-1, according to the new classification system [4], had comparable levels of growth factors to that of the same type of PRP of healthy volunteers. This is despite the systemic effects that burn injury has on the physiology of burn patients. This is relevant additional information to the main RCT, of which the current study was a part [9], that showed that the addition of PRP to the treatment of burn wounds did not result in improved graft take and epithelialization, nor in better scar quality. Only minor beneficial effects in certain subgroups were seen. From the current study, it can be concluded that the lack of substantial clinical effect of the PRP in acute burns does not seem to be explained by a general lack of available growth factors in the PRP of burn patients.

We did find a considerable variation in growth factor concentrations, which is in accordance with the literature on PRP products [7]. More research is required to determine an optimum platelet and growth factor concentration for burns, as well as for other applications. There is some consensus on the minimum platelet counts required in PRP; it is generally advocated that a minimum platelet count of 0.8–1 × 10^6^/μL should be obtained, however, there is no compelling evidence for this. Interestingly enough, we found a correlation between the platelet count in PRP and most of the growth factors measured. This has not always been demonstrated in previous studies [5,6]. The platelet count in PRP did not correlate with the outcome in sub-analyses of the main RCT, of which the current study was a part [9]. Nevertheless, platelet count can potentially be used as a quality control parameter for future research, since it is far more feasible to routinely determine the platelet count in PRP than it is to analyze growth factors. We recommend that further research be done to confirm this finding.

A limitation of this study is that only a small cohort of patients was tested, so the results should be considered preliminary. Furthermore, subgroup analyses were not feasible. The effect of gender and age could not be studied; this may influence the growth factor content, as has recently been suggested [6]. Nor could the effect of the percentage total body surface area burned (TBSA %) or timing of the post-burn injury be clarified. It would also have been very interesting if we had been able to correlate the growth factor content with the clinical outcome in the RCT; however, this was not realistic in this small adjunct study.

In conclusion, in this preliminary study, burn patients have a comparable platelet growth factor content in L-PRPIIB-1 to that of matched healthy volunteers. In accordance with the literature, considerable individual variation in growth factor content was found; however, a correlation between growth factor concentration and platelet count in PRP was seen. 

## 4. Materials and Methods

This study was performed as a sub-study (amendment NL28331.094.09) of a randomized controlled trial (ISRCTN14946762) performed in the burn center of Beverwijk, the Netherlands, which compared autologous platelet-rich plasma with standard treatment for burn wounds [9]. All ethical committee and institutional permissions were obtained to recruit five consecutive patients, who were already included in the main RCT, after additional informed consent had been obtained, between December 2011 and March 2012. The PRP was prepared with the Gravitational Platelet Separation System (GPS-III system, Biomet Merck Biomaterials, Darmstadt, Germany). The instructions of the manufacturer were strictly followed. For details of the trial and preparation methods see previous report [9]. From 27 ml blood from five patients, we prepared PRP with an additional GPS-III mini-kit. We matched the patients by gender and age with five healthy volunteers and also prepared PRP with a GPS-III mini-kit. 

A small amount of PRP and non-citrated whole blood (WB) was collected in an EDTA (ethylene di-amine tetra-acetic acid) tube, and analyzed for baseline measurements, using the Cell-Dyn Sapphire 2 hematology analyzer (Abbott Diagnostics Division, IL, USA). The platelet, erythrocyte, and leukocyte counts were determined.

The PRP was activated with autologous thrombin according to the manufacturer’s protocol (i.e., PRP:thrombin = 10:1) and incubated for one hour at room temperature to mimic clinical application as accurately as possible. Activated PRP was centrifuged (10,000× *g* at 4 °C for 15 min), clots were removed, and supernatants were collected and stored at −80 °C until further analysis. Magnetic bead panel Milliplex MAP kits (EMD Millipore, Billerica, MA, USA) were used to analyze FGF-2, EGF, VEGF, TGFβ-1, TGFβ-2, TGFβ-3, PDGF-AA, and PDGF-BB. Separate kits were used to analyze TGFβ and PDGF. All the kits were used according to the manufacturers’ protocol. The Milliplex MAP kits were measured using Bio-Plex 200 (Bio-Rad, Hercules, CA, USA) and the data were analyzed using Bio-Plex manager software.

For statistical analyses, SPSS statistics 21(IBM) software was used. For the comparison of means, a Mann–Whitney test was used. Correlation was tested with the non-parametric Spearman’s rho test. Significance was set at *p* ≤ 0.05.

## Figures and Tables

**Table 1:** Demographics and hematology results.

	Age	Sex	Post-Burn Day	TBSA * (%)	Platelet Count × 10^3^/μL Whole Blood	Platelet Count × 10^3^/μL Platelet Rich Plasma (PRP)	Leukocyte Count × 10^3^/μL Whole Blood	Leukocyte Count × 10^3^/μL PRP	Erythrocyte Count × 10^6^/μL Whole Blood	Erythrocyte Count × 10^6^/μL PRP
**P1**	35	F	3	16	212	1141	9.2	29.0	3.0	1.2
**P2**	67	F	6	9	467	1792	7.5	30.0	3.8	0.5
**P3**	40	M	10	61	173	1003	8.1	30.0	2.6	1.1
**P4**	61	F	13	12	524	2500	20.3	94.1	2.6	1.9
**P5**	72	M	17	5	343	1326	8.10	29.4	4.0	0.5
**V1**	41	F			282	713	6.3	16.7	4.5	1.5
**V2**	65	F			311	1248	5.8	24.5	4.4	0.7
**V3**	42	M			298	1277	7.5	33.2	4.6	0.9
**V4**	53	F			356	1448	3.8	19.8	4.4	0.7
**V5**	61	M			284	1678	7.5	48.2	4.7	0.9

* TBSA % = percentage total body surface area percentage burned; P = patient; V = Volunteer.

## Supplementary Materials

Supplementary materials can be found at http://www.mdpi.com/1422-0067/20/2/288/s1.
