# Supplementary material for: Growth Factor Quantification of Platelet-Rich Plasma in Burn Patients Compared to Matched Healthy Volunteers
Source: Int J Mol Sci. 2019 Jan 12;20(2):288. doi: 10.3390/ijms20020288 (PMC6358744; doi:10.3390/ijms20020288)
Supplement: Supplementary file 1 [file ijms-20-00288-s001.pdf]

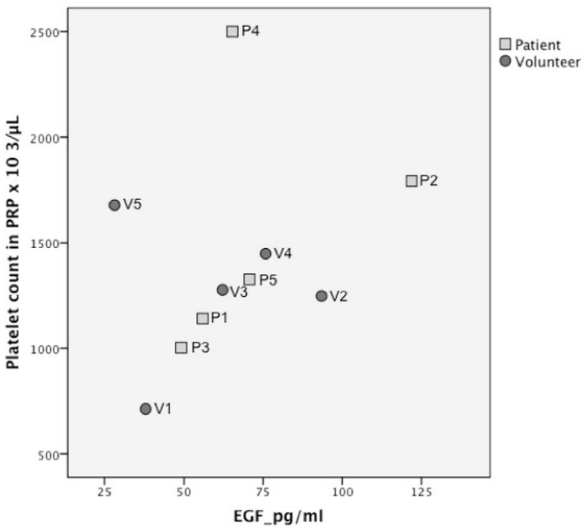

Figure S1. Growth factor quantification per platelet count in PRP for EGF.

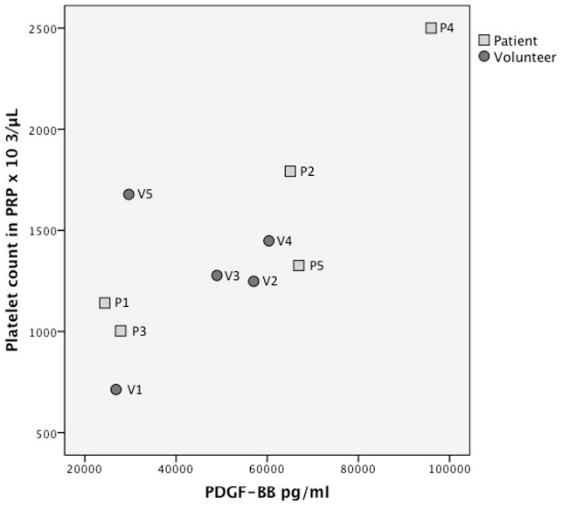

Figure S2. Growth factor quantification per platelet count in PRP for PDGF BB.

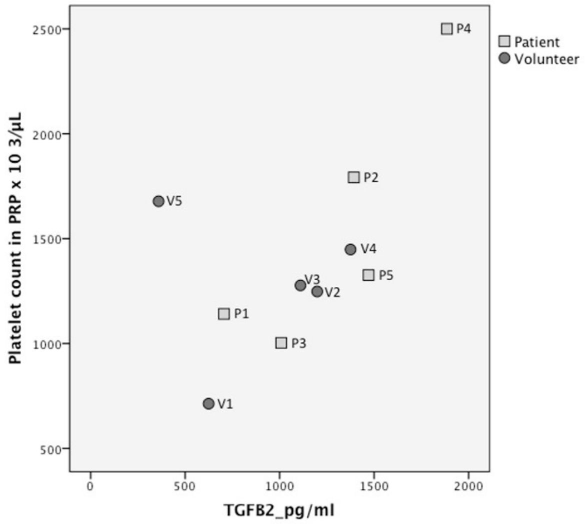

Figure S3. Growth factor quantification per platelet count in PRP for TGFβ2.

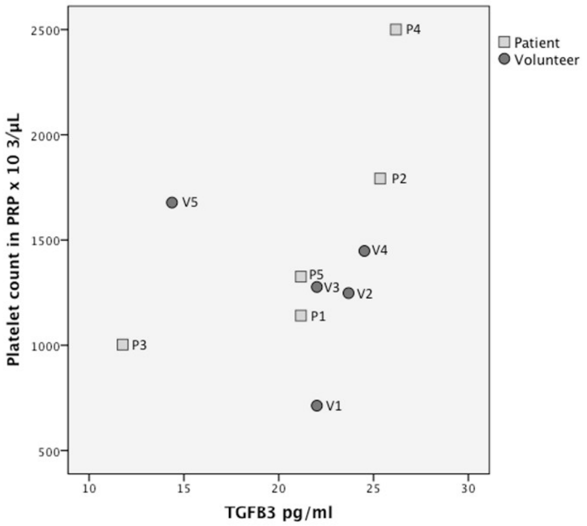

Figure S4. Growth factor quantification per platelet count in PRP for TGFβ3.

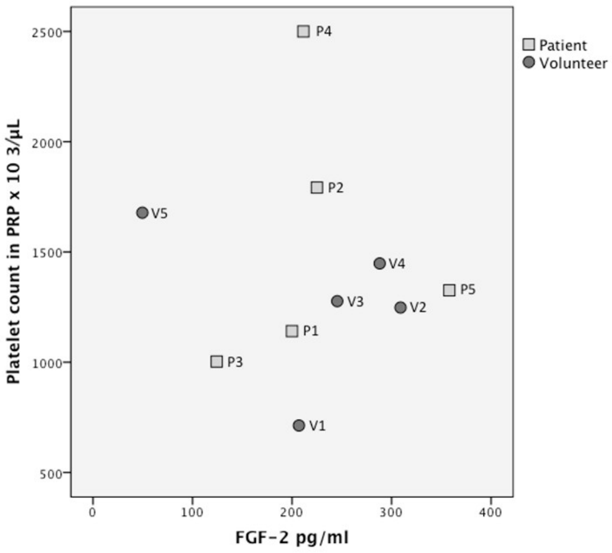

Figure S5. Growth factor quantification per platelet count in PRP for FGF-2.
